# Changes in feral dog populations during the COVID-19 pandemic in Arequipa, Peru

**DOI:** 10.3389/fvets.2025.1666645

**Published:** 2026-01-23

**Authors:** Micaela De la Puente-León, Elvis W. Díaz, Brinkley Raynor Bellotti, Guillermo Porras, Katty Borrini-Mayorí, Olimpia Chuquista-Alcarraz, Valerie A. Paz-Soldán, Michael Z. Levy, Ricardo Castillo-Neyra

**Affiliations:** 1 Zoonotic Disease Research Lab, One Health Unit, School of Public Health and Administration, Universidad Peruana Cayetano Heredia, Lima, Peru; 2 School of Health Sciences, Universidad San Ignacio de Loyola, Lima, Peru; 3 Department of Internal Medicine - Infectious Diseases, Wake Forest University School of Medicine, Winston-Salem, NC, United States; 4 Department of Pathobiology, School of Veterinary Medicine, University of Pennsylvania, Philadelphia, PA, United States; 5 Department of Biostatistics, Epidemiology & Informatics, Perelman School of Medicine at University of Pennsylvania, Philadelphia, PA, United States; 6 Department of Tropical Medicine and Infectious Disease, Celia Scott Weatherhead Tulane University School of Public Health and Tropical Medicine, New Orleans, LA, United States

**Keywords:** dog population management, feral dogs, one health, pandemic, population dynamics, rabies, zoonosis

## Abstract

**Background:**

Feral dogs in Arequipa, Peru, inhabit caves in periurban areas and they may access and rely on organic waste from landfills and livestock from backyard farms. These feral dogs frequently attack small and medium farm animals and occasionally people, posing significant public health risks. Beyond the physical injuries resulting from the attacks, these dogs pose a threat for dog-mediated human rabies as there is active virus transmission in the dog population of Arequipa city. The COVID-19 pandemic restrictions in Arequipa, including restaurant closures, led to a decline in backyard farming and organic waste, thereby reducing food availability for feral dogs.

**Methods:**

We longitudinally (2019–2022) examined the impact of pandemic restrictions on feral dog presence in the periurban areas of Arequipa. Monthly surveys recorded direct and indirect evidence of feral dog presence in caves. An interrupted time series (ITS) analysis evaluated changes in the number of caves with evidence of feral dogs before and after pandemic restrictions. In addition, we conducted in-depth interviews with local farmers to understand the interactions and conflicts between feral dogs and human populations in those periods.

**Results:**

Over 29 months (7 pre-pandemic, 22 during/post-pandemic), an average of 16.42 caves per month showed evidence of feral dog presence, mostly in the form of indirect evidence. Following the pandemic restrictions, the total number of occupied caves decreased by 42% (*p* < 0.010), with a 41% reduction in indirect evidence (*p* = 0.012) and a striking 76% decrease in direct evidence of feral dogs (*p* < 0.001). Farmers described an initial increase in dog attacks immediately following the onset of restrictions, followed by an overall decline as feral dog numbers decreased.

**Conclusion:**

The observed population decline suggests that reduced food availability impacted the local feral dog population. The greater reduction in direct evidence compared to indirect signs indicates a decline in active feral dog presence. These findings describe shifts in feral dog population dynamics during the period of COVID-19 restrictions. The factors driving these changes remain uncertain and could include mortality, migration, or altered behavior. Understanding how food availability and other environmental conditions influence these dynamics is essential for designing interventions that minimize unintended consequences of disease transmission and animal welfare.

## Introduction

A persistent dog rabies epidemic has affected Arequipa city, Peru, at least since 2015 when the rabies virus reintroduction was detected (1). Periurban communities on the outskirts of the city face persistent geographic, economic, and social vulnerabilities that increase their risk of canine rabies, particularly due to limited access to post-exposure prophylaxis following dog bites (1–3). In these same periurban areas, fieldwork conducted in 2019 revealed a previously undocumented issue: feral dogs inhabiting caves in the surrounding landscape (4). Throughout this study, we used the following working definition based on Beaver’s descriptions of dog populations (5): Feral dogs live independently of humans, surviving in natural or remote environments, and generally avoid human contact; they also form packs and are often hunting or scavenging (6–8). These dogs pose a significant threat to public health, livestock, and wildlife in Arequipa (4). Their exclusion from mass vaccination and sterilization campaigns, combined with potential for long-range mobility and high intra-group contact, presents a challenge to reaching the 70% canine vaccination threshold recommended by WHO (or 80% by PAHO) necessary for rabies elimination (9–11).

While data on the ecology of feral dogs in Arequipa remain limited (4), studies in similar urban-adapted wildlife cities (e.g., raccoons, foxes), suggest that feral dogs populations often rely on human-generated food waste, which enables their persistence in periurban environments (12–14). In periurban Arequipa, solid waste is often managed informally (15). Residents may pay waste pickers to collect trash (16), but disposal commonly occurs in nearby water channels (17, 18), geographic features that have also been associated with rabies virus transmission (1). Formal municipal waste collection remains inconsistent due to budgetary and infrastructural limitations (19).

Backyard livestock farming is common in periurban areas of Arequipa. Animals are often fed with organic scraps, obtained through informal supply chains, particularly from restaurants and markets (20, 21). Animal remains and waste from these systems contribute to the diet of feral dogs (4). Disruptions to these food sources—whether due to economic shifts or environmental change—could significantly impact the viability of feral dog populations.

In this study, we leverage the restrictions imposed during the COVID-19 pandemic (22, 23) as a natural experiment to assess the impact of anthropogenic environmental change on feral dog populations. These restrictions substantially reduced the availability of organic waste in landfills and informal animal farms in the periurban areas of Arequipa (24, 25), where cave-dwelling feral dogs are commonly found. Our objective was to evaluate whether there was an association between the timing of the pandemic restrictions and the number of caves exhibiting evidence of feral dog presence in periurban communities. We hypothesize that reduced food availability during the pandemic led to a decline in feral dog activity, as indicated by decreased signs of cave use. The observed association between food scarcity and lower feral dog activity highlights the potential role of improved food waste management in limiting resources that sustain unmanaged feral dog populations, while recognizing that such interventions must be complemented by humane and community-based dog population control strategies.

## Materials and methods

### Study settings

**Figure 1:**
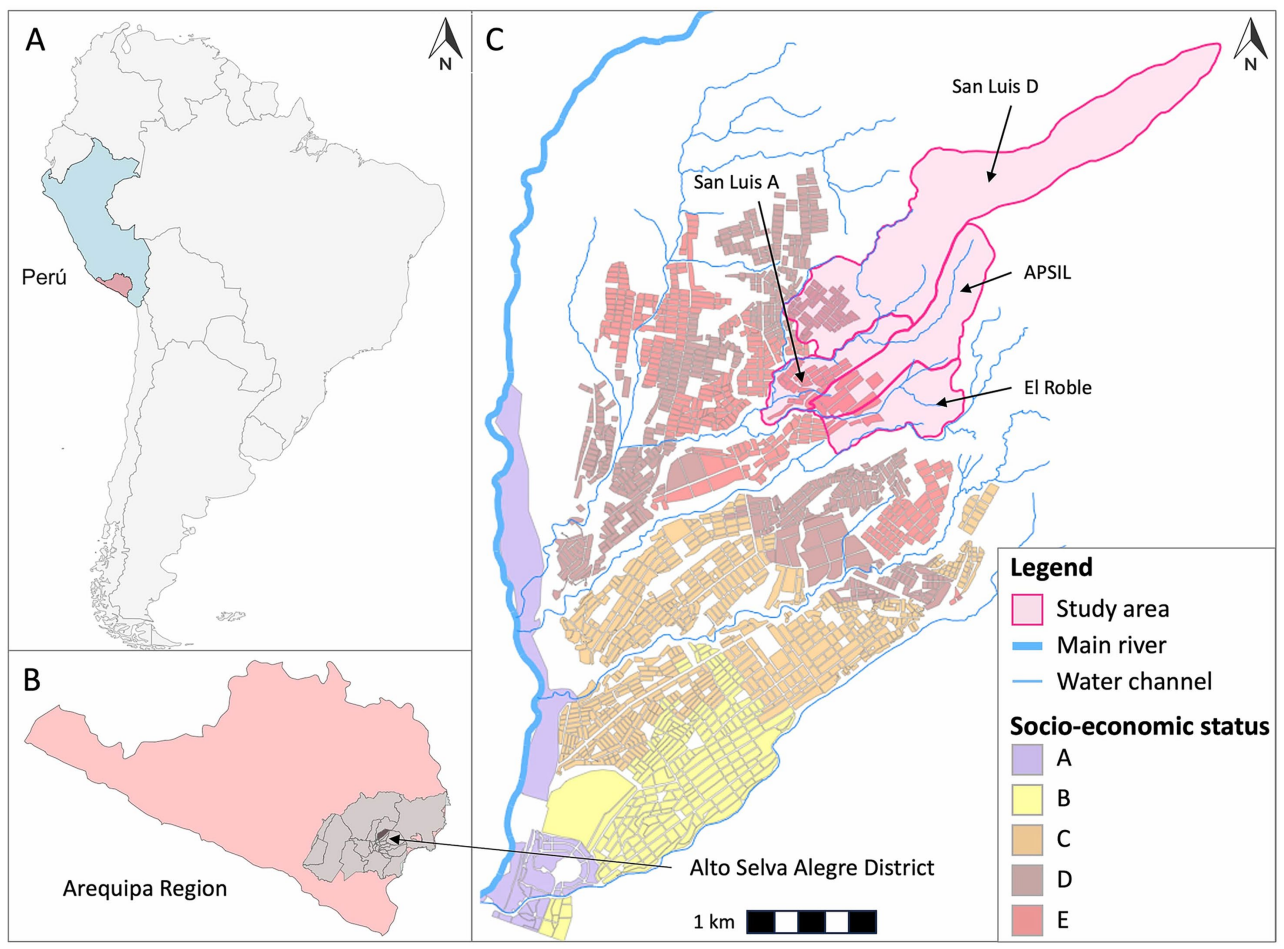
**(A)** Peru, the country where the study was conducted. **(B)** Arequipa region, the canine rabies endemic area in Peru. **(C)** Alto Selva Alegre district, located within the city of Arequipa.

This longitudinal study was conducted from 2019 to 2022 in the periurban areas of the Alto Selva Alegre (ASA) district in Arequipa. These areas were established on the outskirts of the city of Arequipa over the past few decades (26, 27). Periurban areas are characterized by unplanned and rapid growth, often comprised by rural–urban migrants, low SES, very limited infrastructure, and high environmental vulnerability (e.g., poor solid waste management), and overall low socio-economic status (SES). The National Institute of Statistics and Informatics classifies city blocks into five SES levels based on household income and other census-derived indicators (28). In our study area, most city blocks fell within the two lowest SES categories. New settlers usually come together from the same rural town, occupy a discrete and continuous piece of land, and request formal land tenure from the district and city authorities. This discrete geographical area is called a locality, and each locality is engaged in a specific economic activity, such as rock extraction or the raising of domestic farm animals (29, 30). Moreover, each locality is characterized by a different level of backyard livestock keeping. The study population consisted of feral dog and their caves located in four periurban localities: the San Isidro Labrador Pig Farmers Association (APSIL), San Luis Gonzaga Zone A, San Luis Gonzaga Zone D, and El Roble (Figure 1). These caves, which vary in depth, are found in the ground or walls along hillside paths. Feral dogs use them for resting and reproduction (4). Some caves are dug by the dogs themselves, while others are natural formations or landscape features that dogs utilize (4).

### COVID-19 restrictions in Peru

Between March 2020 and March 2022, Peru implemented several phases of COVID-19 restrictions, which varied in intensity and duration, but affected all the country with some consistency in urban areas such as Arequipa city. The initial phase (March–June 2020) involved a strict nationwide lockdown, including curfews, border closures, suspension of public transportation, and the shutdown of schools, restaurants, and markets. Gradual reopening began in mid-2020, followed by alternating periods of partial restrictions through 2021, including nighttime curfews, domestic travel limits between cities, and reduced operating capacity for restaurants. However, some of these restrictions were extended for longer periods in some cities that experience higher COVID-19-associated mortality and one of those cities was Arequipa (31, 32).

In Arequipa, these measures affected the flow of organic waste from restaurants, markets, and informal food vendors, which we consider the key anthropogenic food sources for free-roaming dogs. While some reports indicate a temporary reduction in food waste entering municipal landfills (24), others describe local adaptations such as increased street vending and small-scale food production that may have partially offset these declines (25).

### Cave surveillance

At each study locality, caves were identified by field teams following visible paths created by regular movement of feral dogs. These paths were readily apparent both in the field and on satellite maps. Monthly surveillance visits were conducted to monitor the formation of new caves as well as to collect longitudinal data on the use of caves by feral dogs. Detailed information on the monthly surveys is provided elsewhere (4). Briefly, between September and December 2019, we conducted monthly pedestrian surveys within a 1.6-km^2^ area encompassing the study localities in northeastern ASA to document free-roaming dogs and identify caves used as shelters. A cave was defined as a naturally occurring or canine-modified structure at least 1 meter deep, or sufficiently large to permit entry and shelter, with direct or indirect evidence of dog use; shallower structures with limited evidence of use were mapped as potential sleeping sites. Initial reconnaissance revealed extensive dog-formed trail networks, prompting a pilot comparison of grid-based transects versus dog-trail–based searches. The dog-made trail method was superior to the grid-based method, leading us to adopt that approach for the full survey. We mapped visible dog footpaths using Google Earth (33) and subsequently conducted paired-observer surveys along these trails, recording dog sightings and cave characteristics.

### Cave survey data collection

Field teams collected data through a standardized mobile data collection form developed in the World Veterinary Service (WVS) application (34). The form in the WVS app was designed and piloted by the research team and included fields for cave registration and the presence or absence of various indicators of feral dog activity (Supplement 1).

**Figure 2:**
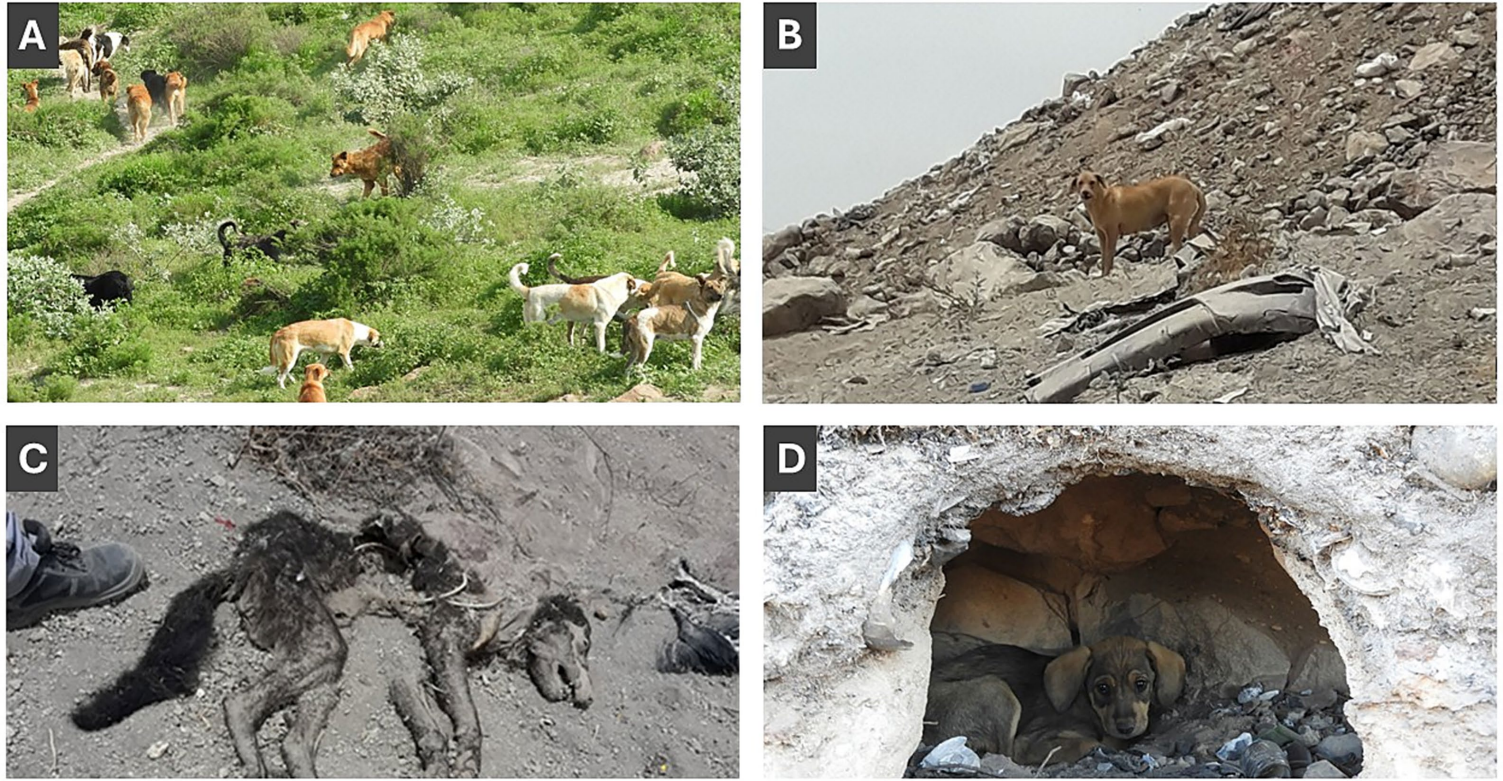
Direct evidence recorded in and around caves: **(A)** Dog packs, **(B)** solitary dogs, **(C)** dead dogs, and **(D)** litters.

**Figure 3:**
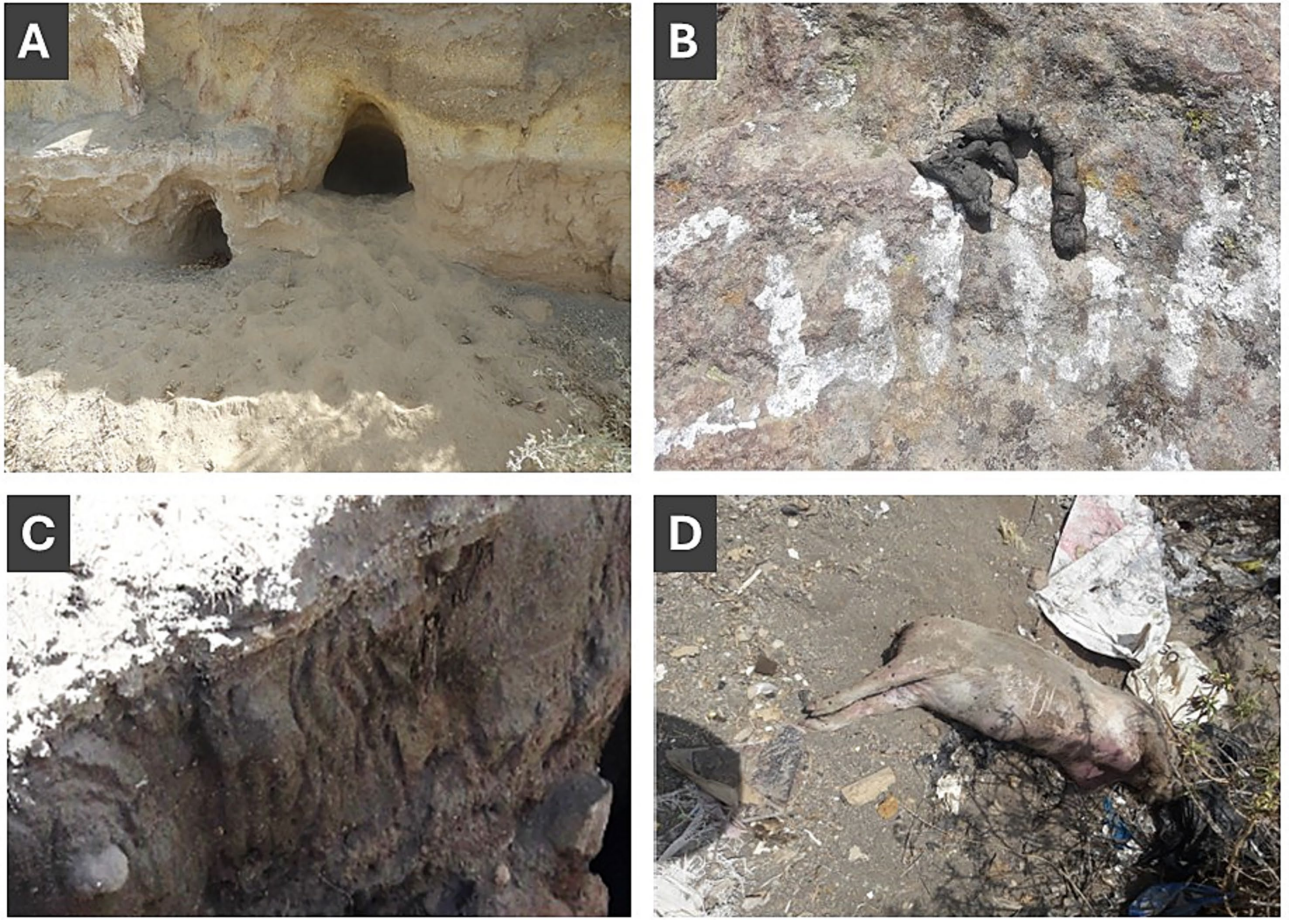
Indirect evidence found in and around cave dogs: **(A)** Paw prints, **(B)** dog feces, **(C)** claw marks in caves, and **(D)** prey carcass of a pig.

For each monthly visit, data on feral dog evidence was recorded. Observed evidence was categorized as direct or indirect. Direct evidence of feral dog presence included: sighting of solitary dogs, dog packs, puppy litters, or dead dogs inside or around the cave opening (Figure 2). Indirect evidence of feral dog presence included canine feces, fresh or desiccated, canine tracks, canine scratch marks, and prey carcases (Figure 3). Additional data were recorded along trails—regardless of cave presence—on dog packs, solitary dogs, and carcasses. No animal handling (e.g., capture, restraint, or sample collection) was conducted in this study. Each cave visit was conducted by two observers with equivalent experience levels, working together to increase detection sensitivity for both caves and animals (4).

### Statistical analysis

The primary outcome analyzed was the count of caves with general evidence of feral dogs per month. Secondary outcomes included the count of caves with direct and indirect evidence, and the number of litters, solitary live dogs, packs, and dead dogs per locality (i.e., all those associated with a cave and those observed not associated with a cave). We compared these primary and secondary outcomes before and after/during the COVID-19 pandemic restrictions. We used Student’s t-tests for mean comparisons and Wilcoxon rank-sum tests for median comparisons. Simple generalized linear models (GLMs) were constructed to explore potential associations of time period (pre- or post- pandemic restrictions) with the primary and secondary outcomes. We used GLMs with a negative binomial family due to overdispersion in the data. In addition, given that a higher number of caves visited increases the likelihood of detecting caves with evidence of feral dogs, we included the number of caves visited each month in each locality as an offset term in our models and evaluated its inclusion using Akaike Information Criterion (AIC; Supplement 2).

After establishing a potential association between the outcomes and the pandemic restrictions, we conducted an interrupted time series (ITS) analysis as our primary analytical framework. ITS is a method used in longitudinal studies to assess whether a response variable changes, and if that change is immediate or progressive, following an intervention. In this study, the onset of COVID-19 restrictions was treated as the intervention, and changes in the number of caves with feral dog evidence were evaluated across this temporal breakpoint. The ITS model tested whether the onset of pandemic restrictions was associated with changes in the number of caves showing evidence of feral dogs before and during the restrictions. ITS used three variables besides the response variable (number of caves with evidence): a continuous variable representing time since the beginning of the study, a continuous variable representing time since the intervention (0 before), and a dichotomous variable indicating whether the time point is before or after the intervention. ITS results indicate whether there was an immediate and/or progressive change in the number of caves with evidence at the start of the restrictions, and whether such a change was statistically significant. The ITS analysis provided information about pre-intervention trends and whether those trends changed following the intervention. For the regression model we used a negative binomial family based on our simple GLMs results. The final parsimonious model was selected by comparing versions of the model with and without the locality variable using log-likelihood estimation. All statistical analyses, tables, and figures were prepared using R version 4.2.0 (35).

### Interviews to farmers

In order to understand the interactions and conflicts between feral dogs and human populations, we conducted in-depth interviews with farmers from periurban communities. These interviews were aimed at capturing lived experiences, farming practices, and community-level responses to the presence of feral dogs. In particular, we sought to explore how changes in food systems, such as the availability of organic waste, fluctuations in livestock production, and informal feeding practices, could contribute to shaping the ecological dynamics of feral dog populations. In the interviews, we explored the effect of the pandemic restrictions on farming activities and the indirect impact on periurban food systems that sustain feral dog populations. By examining how human activities related to food production and waste disposal influence dog behavior and survival, we aimed to generate insights into the socio-ecological drivers of human–dog interactions in rapidly changing periurban environments.

### Interview sampling and recruitment

Purposive sampling was used to select participants across all four study localities. All participants were residents and farmers from periurban neighborhoods. Members of the research team recruited participants through door-to-door visits, during which they obtained contact information (e.g., cell phone numbers). To minimize face-to-face interaction and reduce the risk of COVID-19 transmission, interviews were conducted a few days later by phone.

### Qualitative data collection

Interview guides were developed to explore four main topics: farm animal husbandry, changes in feed and animal prices, interactions with feral dogs, and the impact of the COVID-19 pandemic on farming practices. Two members of the research team conducted the interviews: a Peruvian disease ecology researcher (MDLP, DVM, PhD in Epidemiology) and a Peruvian veterinary epidemiologist (OCA, DVM, MSc).

### Qualitative data management and analysis

All interviews were digitally audio-recorded and transcribed, with detailed notes taken throughout. Centering on the four main topics mentioned above, an inductive coding approach was employed. Initially, the research team immersed themselves in the data to allow key topics to emerge organically, without preconceptions (36). Codes were then developed based on the emerging themes. Data was imported into Dedoose (37) and double-coded by two members of the research team. Any discrepancies in coding were discussed thoroughly until consensus was reached, with new codes added as necessary. All transcripts were re-coded using the finalized coding scheme. To address the primary research question, all data coded under themes related to feral dogs were summarized in tables, and representative quotes were selected for presentation.

## Results

### Study population and cave areas

The study area covered 3.91 km^2^ across the four periurban localities. From September 2019 to March 2022, field teams conducted 9,114 cave observations. During the study period, we identified 38 previously undocumented caves, while 61 of the caves initially recorded had collapsed or otherwise deteriorated and no longer appeared suitable for use as dog shelters. In nearly every monthly visit, at least one cave showed evidence of feral dog activity, except for 2 months in San Luis Gonzaga D-the largest locality but with the fewest farms. On average, 16.42 caves per month showed signs of dog presence, primarily indirect (e.g., paw prints, claw marks, feces), while direct evidence such as live dogs or litters was less frequent (mean = 2.1 caves/month). Paw prints were the most common indicator (99% of positive observations), followed by claw marks (91%).

Across the entire study area, we recorded 46 litters of puppies, 166 dead dogs, 42 solitary adult dogs, and 97 packs. Of the 46 litters, 8 were located within or immediately adjacent to caves, suggesting feral reproduction, while the remaining 38 were found in open areas or near human dwellings, where dogs likely had some level of human contact. In or near caves, we also 25 observations of adult dogs inside caves, and 176 observations of dogs near caves were entrances. The mean litter size was 7 puppies, and the average pack size was 11 dogs.

### Temporal patterns in feral dog evidence

**Figure 4:**
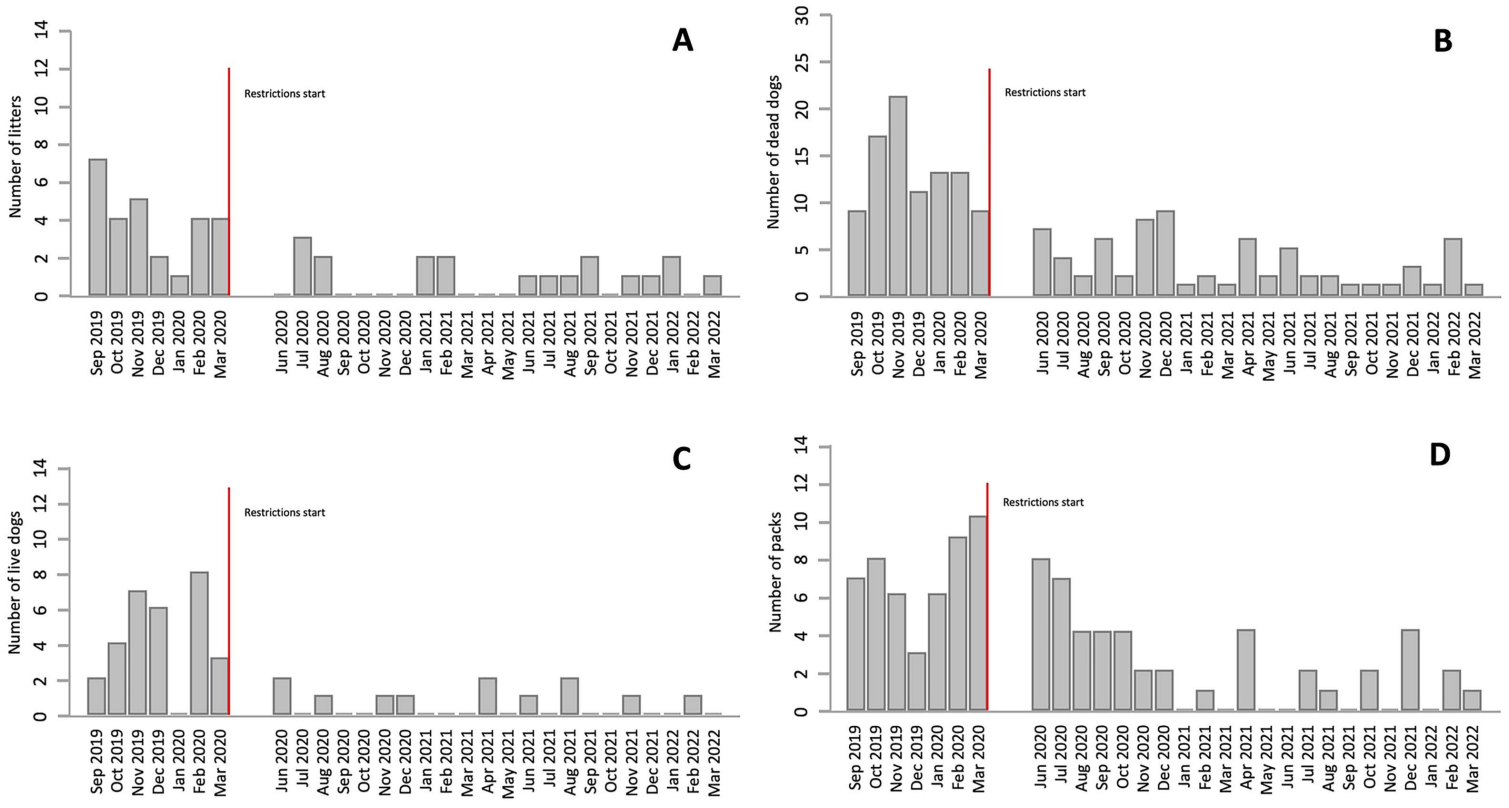
Monthly counts of **(A)** litters, **(B)** dead dogs, **(C)** live dogs, and **(D)** packs. The red vertical line indicates the start of the lockdown due to the COVID-19 pandemic.

Following the onset of COVID-19 restrictions, a statistically significant and sharp decline was observed in the number of caves with general, indirect and direct evidence of feral dog presence, evaluated with simple GLMs. Median monthly caves count with general evidence fell from 31.5 to 10 (*p* < 0.001), while direct evidence declined from 2 to 0 (*p* < 0.001; Table 1). Additional indicators such as litters (*p* < 0.001), solitary dogs (*p* < 0.001), dead dogs (*p* < 0.001), and packs (*p* < 0.001) also showed statistically significant reductions. However, no significant differences were found in the number of puppies per litter (*p* = 0.542) or the number of dogs per pack (*p* = 0.142; Table 2). In addition to measures of central tendency, bar graphs show these trends clearly (Figure 4). A gradual decrease was observed in the number of litters (Figure 4A), dead dogs (Figure 4B), and packs (Figure 4D) at the onset of the pandemic restrictions, along with a substantial drop in the number of live dogs (Figure 4C).

**Table 1:** Number of caves with direct and indirect evidence of feral dogs before and during the COVID-19 pandemic restrictions.

Variables	COVID-19 pandemic restrictions		*p*-value*
	Without restrictions	With restrictions	
Caves with general evidence—monthly median (IQR)	31.50 (22.75–35.75)	10.00 (4.75–19.00)	<0.001
Caves with indirect evidence—monthly median (IQR)	31.50 (22.75–35.75)	10.00 (4.75–19.00)	<0.001
Caves with direct evidence—monthly median (IQR)	2.00 (0.25–6.75)	0.00 (0.00–2.00)	<0.001

*Wilcoxon rank sum test; IQR: Interquartile range.

**Table 2:** Population indicators of feral dogs in caves, trails, and open fields before and during the COVID-19 pandemic restrictions.

Variables	COVID-19 pandemic restrictions		*p*-value*
	Without restrictions	With restrictions	
Litters—monthly median (IQR)	0.50 (0.00–1.00)	0.00 (0.00–0.00)	<0.001
Puppies per litter—monthly median (IQR)	6.00 (3.00–13.00)	5.00 (3.00–6.75)	0.542
Dead dogs—monthly median (IQR)	2.50 (0.00–6.00)	0.00 (0.00–1.00)	<0.001
Solitary live dogs—monthly median (IQR)	0.50 (0.00–2.00)	0.00 (0.00–0.00)	<0.001
Dog packs—monthly median (IQR)	2.00 (0.00–3.00)	0.00 (0.00–1.00)	<0.001
Number of dogs in packs—monthly median (IQR)	11.50 (6.75–18.75)	7.00 (4.00–16.00)	0.142

*Wilcoxon rank sum test; IQR: Interquartile range.

**Figure 5:**
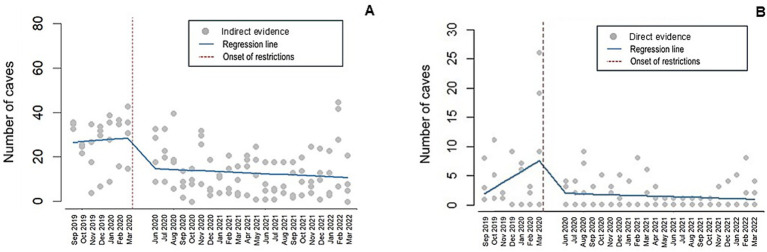
Estimates and trends in ITS analyses before and after COVID-19 pandemic restrictions for **(A)** Indirect evidence and **(B)** Direct evidence of dogs in the cave dogs area.

The ITS models for presence of feral dogs showed a statistically significant decrease in the number of caves with general evidence, indirect evidence, and direct evidence at the onset of COVID-19 restrictions (*p* < 0.010, *p* = 0.012, and *p* = 0.002, respectively; Table 3). Compared to the pre-restriction period, at the onset of restrictions, caves with general evidence decreased by 42%, caves with indirect evidence decreased by 41%, and caves with direct evidence decreased by 76% (Figure 5). Temporal trend variables—“visit number since the start of the study” and “visit number since the onset of restrictions”—were significant for direct evidence. Prior to restrictions, caves with direct evidence increased by 25% per month. After restrictions were implemented, this trend reversed, with the number of caves decreasing by 25% per month. Temporal variables were not statistically significant for general and indirect evidence. In these full multivariable models, locality was also a significant predictor. For all types of evidence, each locality experienced a more pronounced decline following the onset of pandemic restrictions compared to APSIL, the most densely populated human settlement in the study area.

**Table 3:** Parsimonious model for the number of caves with direct evidence of feral dogs in periurban areas of Arequipa.

Variables	Direct evidence*			Indirect evidence*			General evidence*		
	Est	95% CI	*p*-value	Est	95% CI	*p*-value	Est	95% CI	*p*-value
Monthly visit since study beginning	1.25	(1.06–1.49)	0.015	1.02	(0.93–1.11)	0.669	1.02	(0.93–1.11)	0.677
COVID-19 restrictions	0.24	(0.10–0.56)	0.002	0.59	(0.39–0.87)	0.012	0.58	(0.39–0.86)	0.010
Monthly visit since restrictions beginning	0.75	(0.63–0.90)	0.003	0.95	(0.87–1.03)	0.238	0.95	(0.87–1.04)	0.247
Locality									
APSIL	REF	-	-	REF	-	-	REF	-	-
San Luis Gonzaga A	0.24	(0.14–0.41)	<0.001	1.03	(0.79–1.33)	0.845	1.02	(0.89–1.32)	0.876
El Roble	0.13	(0.07–0.23)	<0.001	0.66	(0.51–0.87)	0.002	0.66	(0.51–0.86)	0.002
San Luis Gonzaga D	0.02	(0.001–0.09)	<0.001	0.72	(0.52–0.98)	0.035	0.72	(0.53–0.98)	0.039

*Considering the number of caves visited in each location and each month as the offset.

### Human and feral dog interactions

We recruited 64 farmers during the door-to-door visits; when called, only 41 accepted to participate and were interviewed. All the interviewees raised or had raised farm animals and 20 of them were women. The interviewed farmers mentioned that the pandemic affected the prices of their animals, forcing them to reduce the number of animals they were raising, or, in some cases, to stop raising them altogether. This decline in small-scale animal husbandry not only impacted household income and food security and may have had ecological consequences, such as reduced availability of organic waste that could previously have served as a food source for feral dogs.

*"I used to raise a larger number of animals, mainly pigs. Now, with the pandemic, business has declined and there's not much demand, so the number of animals has decreased."* Man, San Luis Gonzaga A.

*"I used to raise my chickens and ducks, but after the pandemic, that started to disappear."* Woman, APSIL.

Farmers noted that animal farming can serve as a food source for feral dogs, due to inadequate disposal of animal carcasses, which are easily accessible to roaming dogs.

*"Wild dogs are large and hang around the pig farms; sometimes pigs are thrown into the [dry] water channels, and the dogs feed on them."* Man, ASPIL.

The reduction in solid waste during the pandemic, a primary food source for dogs, was associated with increased starvation among feral dog populations. Unexpectedly, despite mostly unfavorable discussions regarding feral dogs, one woman reported that some people actually felt sorry for them:

*“There were other neighbours who would go all the way to the market to bring scraps, intestines, to help feed the dogs because they were completely skinny, they looked pitiful. During the lockdowns there was no garbage to feed the dogs.”* Woman, APSIL.

Multiple dimensions of human–animal conflict emerged during the in-depth interviews with local farmers, mostly describing concerns about safety of themselves, their children, or their farm animals encountering hungry feral dogs. Although some farmers described a short-term rise in predation immediately following the onset of lockdowns, most reported fewer dog attacks later in the pandemic period, consistent with the observed reduction in feral dog evidence. The identified dimensions and their descriptions are presented in Table 4. Interestingly, fecal contamination was not mentioned, despite the widespread presence of feces observed in the study area.

**Table 4:** Dimensions of human-dog conflict in the periurban areas of Arequipa, Peru, 2015.

Dimensions	Quotes
Attacks on livestock	“I mostly stay here on my farm because that’s where my animals are—I have to watch over them, because if I’m not here, there are also wild dogs around that come to eat the animals.” “One day, the neighbor forgot to close the pen and left it open. Around 1 a.m., I heard the pigs screaming. The next morning, I saw that all eight pigs had been eaten.”
Attacks on people	“Our children go to the store to buy things, and sometimes the dogs are hungry… they go with a stick for protection…” “I walk through where the dogs are, even with my child. I have a six-year-old, and we have to pass through there holding a stone in hand for protection.”
Neglect and abandonment	“The dogs were abandoned, they had their puppies who grew up, and so on. And these dogs survive by stealing animals—chickens, sometimes they even come in and eat the cats.”
Scavenging and waste dispersion	“The dogs usually come around here because, as this is a farm, garbage and food waste are sometimes discarded, which attracts wild dogs that then gather in groups.”
Fear and reduced mobility	“You walk down the street and see dogs everywhere.” “I avoid walking through those places because it scares me.”
Animal abuse and culling	“We scare them off with arrows and sticks, and we chase them with rocks so they stop coming around.” “They [other farmers] have tried to poison them, but the dogs do not eat it. A neighbor gave them poison and said the wild dogs did not eat it, but his own puppies ended up dying.”

## Discussion

Since 2015, a canine rabies epidemic has continued in Arequipa, Peru, with 394 confirmed cases reported by April 2025 (38). To effectively address this crisis, it is essential to understand the dynamics of the city’s dog population, particularly feral dogs, which operate outside traditional ownership and exhibit distinct ecological behaviors (6, 7, 13, 39, 40). Although previously unreported, a study published in this special issue confirms the presence of feral dogs in periurban areas. These areas are highly vulnerable to rabies due to high dog bite rates, low vaccination coverage, limited health service access, and a dense population of free-roaming dogs (1, 2, 14, 41, 42). The presence of feral dogs exacerbates these risks. Although feral dog populations are often assumed to be negligible relative to owned free-roaming dogs due to presumed low carrying capacity—defined as the maximum population size a habitat can support—our findings challenge this assumption (43–45). We demonstrate that periurban areas of Arequipa can support substantial feral dog populations, although these populations remain sensitive to fluctuations in local carrying capacity.

Evidence gathered in and around caves confirms that periurban regions on the outskirts of Arequipa city offer high habitat suitability for feral dogs. During the study, over 90% of surveyed caves contained paw prints and scratch marks, while more than half had feces, indicating frequent use. Direct sightings were limited, likely due to the dogs’ avoidance of humans and daytime survey hours, but key indicators such as pups, solitary individuals, packs, and carcasses were documented. The presence of pups points to ongoing reproduction, while dog packs suggest structured social behavior. Globally, feral dogs have been documented in diverse countries like Chile, Brazil, Italy, and India (46, 47). Feral dogs in Arequipa city show similar behavioral traits reported in other countries, such as avoidance of humans and group living (43, 48). Pack sizes ranged from 2 to 22 dogs, with an average of 5, aligning with international data (6–8, 43). Dogs feed primarily on household waste dumped in open fields and supplement their diets by preying on backyard livestock or scavenging from carcasses (4), behaviors that mirror those reported in other countries (47) and have prompted community responses such as fencing, traps, poisoning, and guard dogs, highlighting the economic and social impact of these animals.

The COVID-19 pandemic introduced drastic ecological changes that affected these dogs’ food system, behavior, and interactions with humans. Lockdowns likely reduced organic waste production and disrupted commercial supply chains for animal feed. As a result, backyard farmers became more dependent on household waste to feed livestock, intensifying competition with feral dogs. Farmers initially reported increased livestock predation as food sources suddenly diminished, but over the subsequent months both sightings and attacks declined, consistent with a reduction in feral dog population and carrying capacity. The decline in observed dog activity may reflect increased mortality and reduced recruitment, but emigration is also likely. Because the population was not demographically closed, dogs routinely explored adjacent areas, and pandemic-related restrictions may have accelerated movement toward resource-rich sites such as large dumps. Our prior work shows that one-third of free-roaming dogs travel several kilometers and use dry water channels as ecological corridors (14). Variation in the distribution of these corridors across study sites may partially account for differences in our findings, although other factors, particularly food availability, likely played an important role in shaping this heterogeneity.

The COVID-19 restrictions inadvertently created conditions that reduced the availability of food waste and other resources for feral dogs. While this provided a unique opportunity to study ecological responses under sudden environmental stress, it also revealed the vulnerability and suffering of these neglected animals. Community members frequently described dogs as emaciated and sickly, underscoring the severe welfare implications of food scarcity. It is essential to recognize that population declines driven by starvation are neither humane nor sustainable as management strategies. Instead, these findings emphasize the urgent need for integrated, ethical approaches to feral dog population management—combining vaccination, sterilization, waste management, community engagement, and other humane strategies—to prevent suffering while reducing disease risk.

Feral dogs represent an underrecognized public health challenge in rabies-endemic areas. In Arequipa, 12.4% of periurban residents report being bitten by dogs annually—the highest rate recorded in Latin America (2). Of those bitten, 84% say the dog was unfamiliar, and 73% do not seek medical care. At the time that study was conducted, feral dog populations were unrecognized in the area and the proportion of dog bites associated to feral dogs was not estimated. Even if recognized, in those same areas the proportion of unrestricted owned dogs is also very high making the estimation very challenging (49). Local media frequently report attacks, even fatal, on vulnerable populations, such as children and intoxicated individuals. Additionally, sightings and conflicts involving feral dogs have been reported in multiple districts, including Cayma, Cerro Colorado, Yura, and Mariano Melgar (50–53), indicating that this is not an isolated issue.

Concerningly, current dog rabies vaccination programs focus solely on owned dogs and surveillance activities are restricted to areas with human settlements, excluding feral dog populations (9, 54). Because these dog populations are not recognized and quantified, they are not accounted in the calculations of rabies vaccine coverage causing overestimation of the actual vaccine coverage of dogs at risk (e.g., free-roaming dogs). Our data suggest untapped opportunities to work with feral dogs. Periurban dwellers are already in conflict with these dog populations. This offers a critical opportunity to engage them for passive disease surveillance and pathogen monitoring (55). Importantly, the COVID-19 pandemic demonstrated how quickly environmental changes can shift the dynamics of feral dog populations. This reinforces the need for comprehensive public policies, particularly around solid waste management, not just as a sanitation issue, but as a vital component of zoonotic disease prevention and human-wildlife coexistence (56, 57).

The persistence of rabies in Arequipa, the ecological adaptability of feral dogs, and their increasing interactions with humans and wildlife call for an urgent, integrated response. Effective interventions must include targeted surveillance, inclusion of feral dogs in rabies control efforts, and robust environmental management to reduce the conditions that allow these populations to thrive.

Our study had some challenges. Local farmers reported observing dogs with poor body condition, but we did not collect quantitative measures on dogs’ body condition during the study. The sensory abilities and behavioral nature of feral dogs allow them to detect human presence from a distance, potentially causing them to flee or leave their shelters before detection. Due to this limitation, we supplemented our observations with indirect evidence of dog presence, including feces, tracks, scratch marks, and nearby food and water sources. These indirect signs varied in reliability; for instance, fresh feces and tracks are considered more dependable indicators, particularly under the climatic conditions of Arequipa, where such evidence rapidly degrades. While indirect evidence helps infer site use by feral dogs, it does not provide precise information about the timing of their presence, making direct observations more conclusive. Similarly, the identification of deceased dogs within the study area did not allow for confirmation of their feral status, as they could have been unowned roaming dogs, community dogs, or even owned dogs with outdoor access. Consequently, the actual mortality of feral dogs may be overestimated. However, this potential overestimation would have affected both pre-pandemic and pandemic-era data equally. During our visits, we noticed that some caves had been destroyed which could cause overestimation of occupancy. The turnover in cave availability suggests a dynamic shelter landscape that may reflect broader environmental and socioecological changes occurring in the area. For example, caves could be degraded or destroyed through routine human and livestock activities or become unsuitable for dog shelter as a result of rainfall and eolian erosion. We adjust for that by using the number of caves at each visit as an offset. Although our study implies interaction of feral dogs and humans and other animals base on proximity, it did not permit evaluation of interspecific interactions, such as with owned dogs or native wildlife like foxes, before and during the pandemic.

The presence of feral dog increases the vulnerability of local human communities, especially in a region that already reports the highest rates of dog bites in Latin America, has extremely low socio-economic status, and lacks access to healthcare, including PEP. Based on our findings, environmental management emerges as a key factor in modulating the interaction between human settlements and feral dog populations; improved practices around animal husbandry and solid waste management could offer an efficacious and sustainable approach to reduce the problems associated with feral dogs. However, despite evidence suggesting both mortality and migration during the pandemic, the relative contribution of each process to the observed reduction in occupied caves remains unclear. If migration is occurring, it raises concerns about the potential spread of pathogens such as the rabies virus into rabies-free zones, either urban or the wilds, as well as the ecological impacts of interspecies interactions and predation on native wildlife by feral dogs. Given the complexity of the periurban ecology, any environment-based intervention intended to reduce the carrying capacity for feral dogs should be accompanied by monitoring of intended and also unintended consequences. It is critical to integrate these newly identified dog subpopulations in the dog rabies control program to move toward the goal of eliminating dog-mediated human rabies by 2030 (58).

## Data Availability

The original contributions presented in the study are included in the article/Supplementary material, and further inquiries can be directed to the corresponding author.
