# Supplementary material for: Changes in feral dog populations during the COVID-19 pandemic in Arequipa, Peru
Source: Front Vet Sci. 2026 Jan 23;12:1666645. doi: 10.3389/fvets.2025.1666645 (PMC12875945; doi:10.3389/fvets.2025.1666645)
Supplement: Supplementary file 1 [file Data_Sheet_1.pdf]

The impact of COVID-19 pandemic restrictions on feral dogs in Arequipa, Peru

Supplement 1. Form for Recording Information on Cave Area Visits in the WVS Application

Nro	Pregunta	Tipo de pregunta		Respuestas
	**PREGUNTAS GENERALES**			
1	Zona	DropDown List		
			☐	APSIL
			☐	San Luis zona A
			☐	San Luis zona D
			☐	El Roble
			☐	Otra zona
	Completar 1a si marcó "Otra zona" en la pregunta 1.			
1a	Especificar la zona	Text		______
2	Fecha	Datepicker		______
3	Hora	Timepicker		______
4	Tipo de entrada			
			☐	Características del sitio
			☐	Cueva
			☐	Perro muerto
			☐	Crías de perros
			☐	Perro vivo
			☐	Jauría
			☐	Observaciones o hallazgos adicionales
	**PERRO VIVO**			

Si marcó "PERRO VIVO" en la pregunta 4, completar las preguntas 5-14.

5	Foto 1 del perro vivo	Image	
6	Foto 2 del perro vivo	Image	
7	Tamaño del perro	Radio Button	
			☐ Pequeño
			☐ Mediano
			☐ Grande
8	Edad en años	Numeric	
9	Edad en meses	Numeric	
10	Condición corporal	DropDown List	
			☐ 1 (caquéctico)
			☐ 2 (bajo peso)
			☐ 3 (peso ideal)
			☐ 4 (sobrepeso)
			☐ 5 (obeso)
			☐ No puedo determinarlo
11	Calidad del pelaje	DropDown List	
			☐ Regular
			☐ Brilloso
			☐ No puedo determinarlo
			☐ Descuidado
12	Raza aparente	DropDown List	
			☐ Pura
			☐ Características distintivas
			☐ Mestizo o criollo
			☐ No puedo determinarlo

Completar 12a si marcó "PURA" en la pregunta 12.

12a	Especificar raza	DropDown List	
			☐ Beagle
			☐ Collie

			☐ Boxer
			☐ Bull Terrier
			☐ Bulldog
			☐ Caniche/Poodle
			☐ Carlino/Pug
			☐ Chihuahua
			☐ Chow Chow
			☐ Cocker
			☐ Dálmata
			☐ Dobermann
			☐ Dogo
			☐ Golden Retriever
			☐ Labrador
			☐ Pastor Alemán
			☐ Pekinés
			☐ Pitbull
			☐ Rottweiler
			☐ Samoyedo
			☐ San Bernardo
			☐ Husky Siberiano
			☐ Otro
	Completar 12b si marcó "OTRO" en la pregunta 12a.		
12b	Especificar otra raza	Textbox	______
	Completar 12c si marcó CARACTERÍSTICAS DISTINTIVAS en la pregunta 12.		
12c	Especificar raza	DropDown List	
			☐ Beagle
			☐ Collie
			☐ Boxer
			☐ Bull Terrier
			☐ Bulldog

			☐ Caniche/Poodle
			☐ Carlino/Pug
			☐ Chihuahua
			☐ Chow Chow
			☐ Cocker
			☐ Dálmata
			☐ Dobermann
			☐ Dogo
			☐ Golden Retriever
			☐ Labrador
			☐ Pastor Aleán
			☐ Pekinés
			☐ Pitbull
			☐ Rottweiler
			☐ Samoyedo
			☐ San Bernardo
			☐ Husky Siberiano
			☐ Otro
	Completar 12d si marcó "OTRO" en pregunta 12c.		
12d	Especificar otra raza	Textbox	
13	¿El perro está asociado a alguna casa?	Radio Button	
			☐ Sí
			☐ No
14	Observaciones adicionales	Textbox	

JAURÍA

Si marcó "JAURÍA" en la pregunta 4, completar de la pregunta 15-20.

15	Foto 1 de la jauría	Image	
16	Foto 2 de la jauría	Image	

17	¿Cuántos perros hay?	Numeric	
18	¿Dónde están los perros?	Radio Button	
			Con una casa
			Deambulando
			Otro
	Completar 18a si marcó "OTRO" en la pregunta 18.		
18a	Especificar	Textbox	
19	¿Qué están haciendo los perros?	DropDown List	
			Caminando
			Buscando comida
			Durmiendo
			Peleando
			Cuidando casa
			Otro
	Completar 19a si marcó "OTRO" en la pregunta 19.		
19a	Especificar	Textbox	
20	Observaciones adicionales	Textbox	

CRÍAS DE PERROS

Si marcó "CRÍAS DE PERROS" en la pregunta 4, completar de la pregunta 21-27.

21	Foto 1 de camada	Image	
22	Foto 2 de camada	Image	
23	Número de cachorros	Numeric	
24	Edad de los cachorros	Radio Button	
			0-10 días
			11 días - 3 semanas
			3-8 semanas
			> 8 semanas

25	¿Están en una cueva?	Radio Button	
			☐ Sí
			☐ No
26	¿La mamá está cerca?	Radio Button	
			☐ Sí
			☐ No
27	Observaciones adicionales	Textbox	______

PERRO MUERTO

Si marcó "PERRO MUERTO" en la pregunta 4, completar de la pregunta 28-37.

28	Foto 1 de perro muerto	Image	
29	Foto 2 de perro muerto	Image	
30	Estado de descomposición	DropDown List	
	**Definiciones de los estados de descomposición: Fresco (sin decoloración de piel o actividad de insectos), Temprana (decoloración gris o verdosa de la piel, hinchazón, pérdida de pelo), Avanzada (descomposición húmeda de los tejidos, alta actividad de insectos, exposición de huesos de menos de la mitad del cuerpo, momificación), Casi extrema (huesos con algunos fluidos o tejido cubriendo menos de la mitad del cuerpo, huesos secos) y Extrema (esqueleto)**		
			☐ Fresco
			☐ Temprana
			☐ Avanzada
			☐ Extrema
			☐ Casi extrema
31	¿Su cadáver ha sido aparentemente descartado por personas?	Radio Button	
			☐ Sí
			☐ No
32	¿Su cabeza ha sido removida?	Radio Button	
			☐ Sí

			☐ No
33	¿Hemos colectado muestra de cerebro?	Radio Button	
			☐ Sí
			☐ No
33a	Código de la muestra colectada	Textbox	____
34	¿Hemos marcado al perro?	Radio Button	
			☐ Sí
			☐ No
34a	¿Con qué hemos marcado al perro?	Textbox	____
35	Escriba el código de la última cueva visitada	Textbox	____
36	¿A cuántos metros aproximadamente se encuentra de la última cueva visitada?	Numeric	____
37	Observaciones adicionales	Textbox	____

CUEVA

Si marcó "CUEVA" en la pregunta 4, completar de la pregunta 38-46.

38	Número de cueva		____
39	¿Esta cueva existe?	Multi Select	
			☐ Sí, existe
			☐ No, está derrumbada
			☐ No existe
40	Foto 1 de la cueva	Image	
41	Foto 2 de la cueva	Image	
42	¿Hay evidencia de que los perros usen la cueva?	Radio Button	
			☐ Sí
			☐ No
			☐ No estoy seguro

Completar 42a si marcó "NO ESTOY SEGURO" en la pregunta 42.

42a	Especificar por qué no estás seguro	Textbox	____
43	Marca la posible evidencia de que los perros usen la cueva	Multi Select	
			☐ Huellas
			☐ Arañazos

- [ ] Heces frescas
- [ ] Heces secas
- [ ] Camada
- [ ] Perro(s) en cueva
- [ ] Perros cerca a la cueva
- [ ] Restos de animales
- [ ] Fuente de comida
- [ ] No hay evidencia
- [ ] Otro
- [ ] Fuente de agua

Completar 43a si marcó "OTRO" en la pregunta 43.

43a	Especificar otra evidencia	Textbox	
44	Descripción de los restos animales	Multi Select	

- [ ] Huesos
- [ ] Pelo
- [ ] Carcasa fresca
- [ ] Oveja o cabra
- [ ] Pollo
- [ ] Perro
- [ ] Otro

Completar 44a si marcó "OTRO" en la pregunta 44.

44a	Especificar otro resto de animal	Textbox	

En la pregunta siguiente solo marcar la opción "Perro" si el perro está siendo consumido como comida de otros canes. De lo contrario (si es un perro muerto que no es fuente de comida), no marcar esa opción.

**45**	Especie animal probable	Multi Select	

- [ ] Pollo
- [ ] Oveja
- [ ] Cabra
- [ ] Perro
- [ ] Gato

			☐	Cuy
			☐	Otro
			☐	No puedo determinarlo
	Completar 45a si marcó "OTRO" en la pregunta 45.			
45a	Especificar animal probable	Textbox	___	
46	Observaciones adicionales	Textbox	___	
	**CARACTERÍSTICAS DEL SITIO**			
	Si marcó "CARACTERÍSTICAS DEL SITIO" en la pregunta 4, completar de la pregunta 47-51.			
**47**	¿Qué has observado de los siguientes en este sitio?	Multi Select		
			☐	Fuente de agua
			☐	Basura
			☐	Recipiente de agua
			☐	Recipiente de comida
48	Número de perros	Numeric	___	
49	Número de jaurías	Numeric	___	
50	¿Cuál es el tipo de suelo?	DropDown List		
			☐	Arenoso
			☐	Rocoso
			☐	Arcilloso
			☐	Agrícola
			☐	Otro
	Completar 50a si marcó "OTRO" en la pregunta 50.			
50a	Especificar tipo de suelo	Textbox	___	
51	Observaciones adicionales	Textbox	___	
	**HALLAZGOS ADICIONALES**			
	Si marcó "HALLAZGOS ADICIONALES" en la pregunta 4, completar de la pregunta 47-51.			

52	Foto 1	Image
53	Describir	Textbox

Supplement 2.

Model	Without offset	With offset
**Estimate (95% CI)**	-0.76 (-1.09 - -0.45)	-0.90 (-1.16 - -0.66)
**Standard Error**	0.162	0.128
**p value**	2.34E-06	1.38E-12
**AIC**	844.4	790.16
**BIC**	852.61	798.37

Comparison between two generalized linear models (GLMs): one simple model and another including the number of visited caves as an offset. Both models use the number of caves with general evidence as the response variable and the presence of restrictions as the explanatory variable. The AIC comparison shows that the model with the offset provides a substantially better fit, with a difference of 50 AIC units in favor of the offset model. According to this model, the log of the number of caves with general evidence is 0.90 units lower under restriction conditions compared to before the restrictions.
Similar results were observed when using the number of caves with direct evidence and the number of caves with indirect evidence as response variables.
